# Following the Preclinical Data: Leveraging the Abscopal Effect More Efficaciously

**DOI:** 10.3389/fonc.2017.00066

**Published:** 2017-04-07

**Authors:** Wilfred Ngwa, Zi Ouyang

**Affiliations:** ^1^Radiation Oncology, Brigham and Women’s Hospital, Boston, MA, USA; ^2^Physics and Applied Physics, University of Massachusetts Lowell, Lowell, MA, USA

**Keywords:** abscopal effect, radiotherapy, immunoadjuvants, metastasis, immunoregulation

## Abstract

Radiotherapy is employed in the treatment of over 50% of cancer patients. However, this therapy approach is limited to mainly treating localized disease. In 1953, Mole described the remarkable abscopal effect, whereby, localized radiotherapy of a patient’s primary tumor might engender regression of cancer at distant sites, which were not irradiated. Current consensus is that if the abscopal effect can be efficaciously leveraged, it would transform the field of radiation oncology, extending the use of radiotherapy to treatment of both localized and metastatic disease. A close examination of the literature on the abscopal effect proffers a disruptive new hypothesis for consideration in future clinical trials. This hypothesis is that generating a subcutaneous human tumor autograft as the primary tumor may be a more efficacious approach to prime the abscopal effect. Following the preclinical data, the merits and demerits of such an approach are examined in this article.

## Introduction

Radiotherapy is a crucial component of cancer care used in the treatment of over 50% of cancer patients either alone or in combination with other treatments. However, this therapy approach is generally prescribed for treatment of localized disease. In 1953, Mole described the abscopal effect (1) whereby localized radiotherapy at one site might engender tumor regression at distant untreated sites. Unfortunately, the abscopal responses to radiotherapy alone are rare (2). In fact, since Mole’s report, only a limited number of cases on the abscopal effect have been reported when using radiotherapy alone. However, it soon became apparent that if this potent effect was efficaciously leveraged, it could transform radiotherapy practice. It would significantly extend the use of radiotherapy to treating both localized and metastatic disease. The impact would be major since cancer metastasis accounts for over 90% of all cancer-associated suffering and death.

A more modern understanding of the abscopal effect is that it is an immune modulation effect of radiotherapy. In a landmark study in 2004, the abscopal effect was first connected to mechanisms involving the immune system (3). The study showed that the effect could not occur in T cell-deficient mice. Subsequent studies have corroborated this, and it has become apparent that, in some cases, radiotherapy may successfully immunize a patient against cancer, converting the irradiated tumor into an *in situ* vaccine (4). In other words, the patient’s immune system may be triggered for a systemic rejection of cancer by treating a tumor lesion locally. The *modus operandi* for such *in situ* vaccination is that the radiotherapy beam first inflicts damage on the cancer cells, eliciting phenotypic changes, and the release of neoantigens (5, 6). The neoantigens can be taken up by antigen-presenting cells (APCs) with the unique ability to process antigenic proteins into suitable peptide fragments, to incorporate them into MHC class I and II molecules, and to present them to T cells. It has also been shown that cancer cell surface expression of MHC class I molecules increases after radiotherapy and in a dose-dependent manner, leading to the recognition of irradiated cells by cytotoxic T lymphocytes (7). Altogether, these and other studies support the fact that the abscopal effect is immune-mediated with direct involvement of T cells.

**Figure 1:**
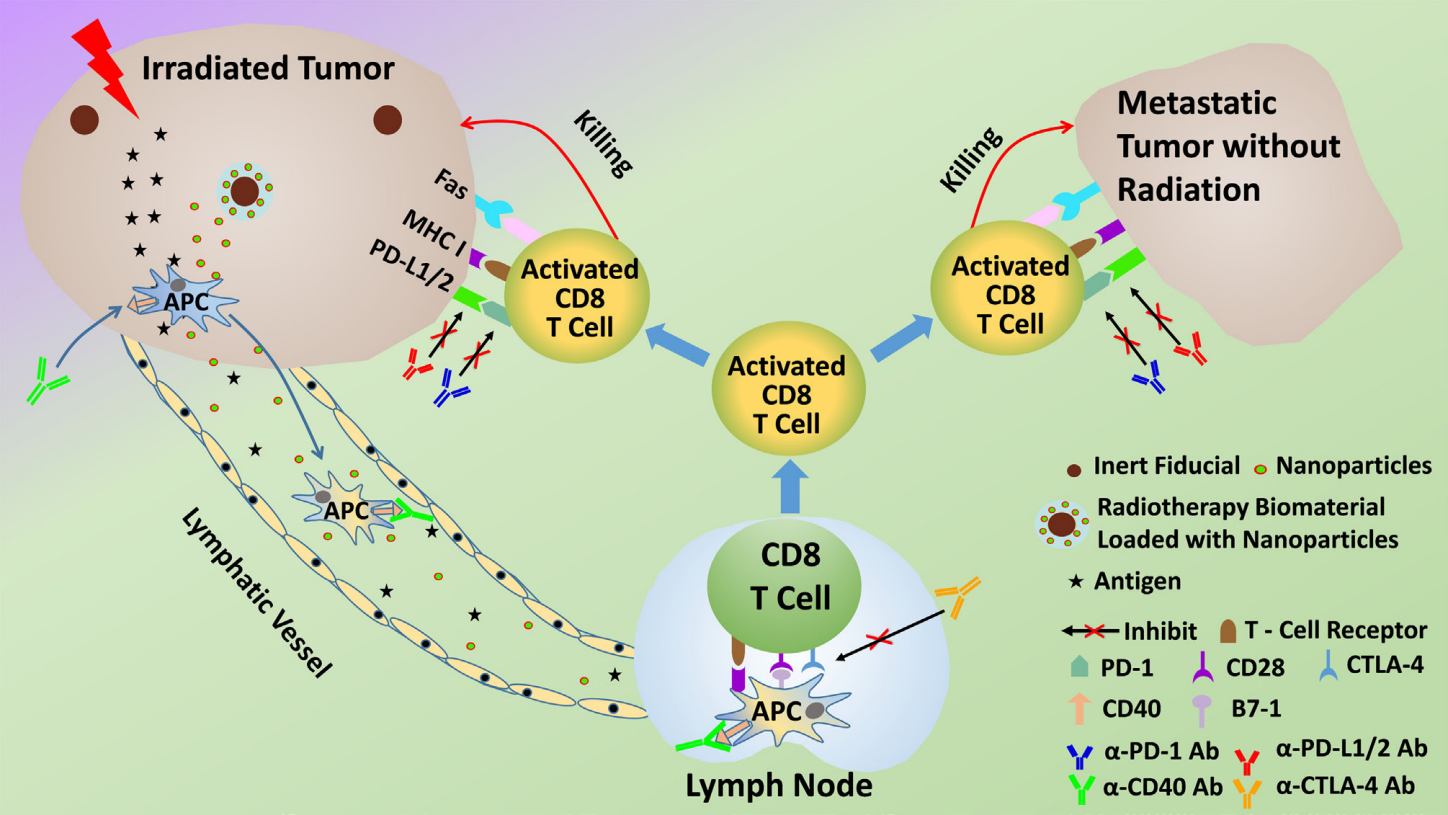
**Schematic of potential *modus operandi* for combining radiotherapy with immunoadjuvants to prime the abscopal effect more efficaciously**.

Given the ability of radiotherapy to convert tumors into an *in situ* vaccine, it follows that the addition of appropriate immunoadjuvants could enhance or prime the immune-mediated abscopal effect and increase response rates. The abscopal effect should really be considered as a product of the multimodal cancer treatment. In fact, many preclinical studies that have demonstrated effective abscopal responses employ immunoadjuvants (Table 1). These preclinical studies have provided justification for clinical trials (8, 9), where abscopal responses are detected in patients treated with radiation therapy and immunoadjuvants, for different indications. In this approach, illustrated in Figure 1, the immunoadjuvants can be employed to target and enhance different aspects of the abscopal effect process. For example, anti-CD40 can be employed to enhance activation of APCs (9), granulocyte macrophage colony-stimulating factor (GM-CSF) can be used to increase the percentage of APCs, while anti-CTLA4 or PD-1 can act as immune checkpoint inhibitors, enhancing T cell action on the tumor cells (9, 10).

**Table 1:** **Preclinical studies demonstrating the abscopal effect when using radiotherapy in conjunction with immunoadjuvants**.

Tumor type	Irradiated site; dose	Immunoadjuvant; dose	Reference
Lewis lung carcinoma	Subcutaneous flank; 6 Gy	Anti-CD40; 20 μg	Ngwa et al. (11)
67NR mammary carcinoma	Subcutaneous; 3 Gy × 8 Gy	Fms-like tyrosine kinase receptor 3 ligand (Flt3-L); 10 μg × 10	Habets et al. (12)
TUBO mammary/MCA38 colon	Subcutaneous flank; 12 Gy	Anti-PD-L1; 200 μg × 4	Deng et al. (13)
FM3A mammary	Subcutaneous flank; 6 Gy	ECI301; 600 ng	Kanegasaki et al. (14)
Colon26	Subcutaneous flank; 20 Gy	IL-2; 20,000 U in 0.1 mL of PBS	Yasuda et al. (15)
TSA mammary/MCA38 colon	Subcutaneous flank; 20, 24, and 30 Gy	9H10; 200 μg × 3	Dewan et al. (16)
Colon26/MethA sarcoma/LLC	Subcutaneous flank; 6 Gy	ECI301, 2 μg × 3	Shiraishi et al. (17)
SCC VII	Subcutaneous femur; 4–10 Gy	DC	Akutsu et al. (18)
4T1 mammary	Subcutaneous flank; 12–24 Gy	9H10	Demaria et al. (19)
67NR mammary	Subcutaneous flank, flank; 2–6 Gy	Flt3-L	Demaria et al. (3)
D5 melanoma/MCA 205 sarcoma MethA	Subcutaneous flank; 42.5 Gy	DC	Teitz-Tennenbaum et al. (20)
C3 cervical/sarcoma	Subcutaneous hind leg; 30–50 Gy	DC	Nikitina and Gabrilovich (21)
LCC	Subcutaneous foot; 60 Gy	Flt3-L	Chakravarty et al. (22)

*LCC, Lewis lung carcinoma; DCs, dendritic cells; SCC, squamous cell carcinoma*.

In a recent prospective clinical trial, using GM-CSF, abscopal responses occurred in 11 of 41 accrued patients (8). In another study, Grimaldi et al. reported a 52% abscopal response rate among 21 patients with melanoma who progressed after receiving the immunoadjuvant ipilimumab (anti-CTLA4) during palliative radiotherapy (23). Noteworthy, in this report, was an indication that a local response was a prerequisite for priming an abscopal effect. Furthermore, a 2015 review article by Reynders et al. (24) described 23 case reports and 13 preclinical studies on the abscopal effect. They observed that 11 of the 13 preclinical studies used immunoadjuvants to achieve an abscopal response. Altogether, these findings strongly suggest that a combination of radiotherapy with immunoadjuvants improves abscopal response rates compared to using radiotherapy alone.

**Figure 2:**
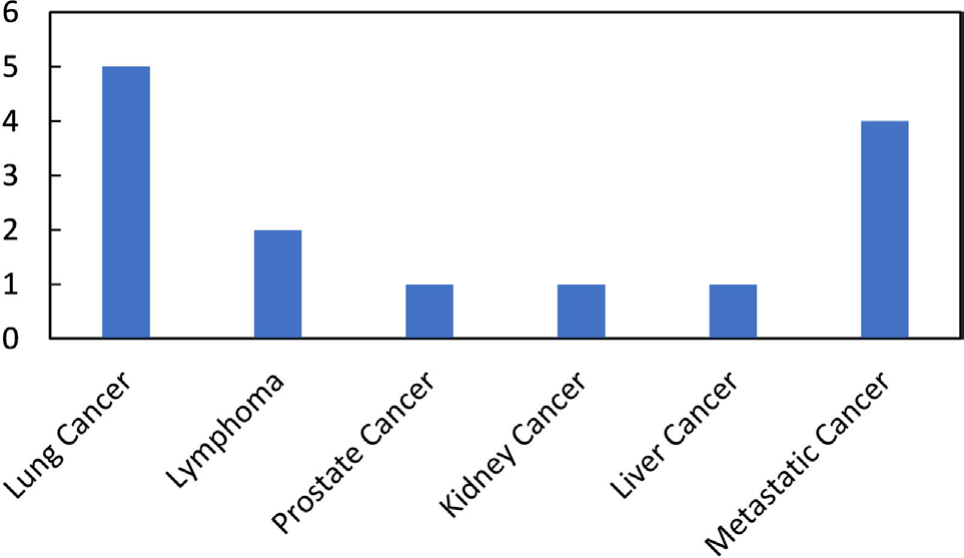
**Number of ongoing clinical trials on combined radiotherapy and immunotherapy per disease site (summarized from the Table 2)**.

However, the cure rates achieved with these combinations in clinical trials have not been as high, or the combinations as effective, as expected from preclinical studies. Hence, these studies have galvanized many ongoing studies investigating approaches that can more efficaciously use immunoadjuvants to prime the abscopal effect and increase cure rates for more patients (9, 25, 26). Besides, many current clinical trials (Table 2; Figure 2) are also focused on treating cancer patients with combined radiotherapy and immunotherapy. Although these studies do not necessarily investigate the abscopal effect, their results will contribute to useful insights for clinical abscopal treatment study.

**Table 2:** **A partial list of current clinical trials that study combined radiotherapy and immunotherapy (based on http://clinicaltrials.gov)**.

Identifier	Study title
NCT03035890	Hypofractionated radiation therapy to improve immunotherapy response in non-small cell lung cancer
NCT02710643	“MIRO” molecularly oriented immuno-radiotherapy (FIL_MIRO)
NCT02579005	Radio-immuno-modulation in lung cancer
NCT02864615	Safety and preliminary efficacy of stereotactic body radiation therapy (SBRT) in patients with metastatic RCC treated with targeted or IO therapy
NCT02463994	A pilot study of MPDL3280A and HIGRT in metastatic none small cell lung cancer
NCT02839265	FLT3 ligand immunotherapy and stereotactic radiotherapy for advanced non-small cell lung cancer (FLT3)
NCT02710253	Phase II trial of salvage radiation therapy to induce systemic disease regression after progression on systemic immunotherapy
NCT03042156	Immunotherapy and palliative radiotherapy combined in patients with advanced malignancy
NCT02843165	Checkpoint blockade immunotherapy combined with stereotactic body radiation in advanced metastatic disease
NCT01436968	Phase 3 study of ProstAtak^®^ immunotherapy with standard radiation therapy for localized prostate cancer (PrTK03)
NCT02677155	Sequential intranodal immunotherapy combined with anti-PD1 (pembrolizumab) in follicular lymphoma (Lymvac-2)
NCT02239900	Ipilimumab and SBRT in advanced solid tumors

## Rationale and Merits for Using Subcutaneous Autografts

### Following the Preclinical Data

As highlighted in Table 1, a considerable amount of preclinical work, which has successfully combined radiotherapy with immunoadjuvants, has involved the generation of subcutaneous tumors as the primary tumor in small animal models, and then priming the subcutaneous tumors to engender the abscopal effect. As an example, in the landmark study in 2004 (3), mice bearing subcutaneous syngeneic mammary carcinoma in both flanks were treated with the immunoadjuvant Fms-like tyrosine kinase receptor 3 ligand (Flt3-L) during radiotherapy. Flt3-L was employed to enhance the number of available APCs, which can take up the antigens after tumor irradiation. Flt3-L was administered after local radiation therapy to only 1 of the 2 tumors. The second non-irradiated tumor was used as indicator of the abscopal effect. Radiotherapy alone led to growth delay exclusively of the irradiated primary tumor. However, the non-irradiated tumor was also impaired by the combination of radiotherapy and Flt3-L.

It is generally thought that the relative success of combining radiotherapy with immunoadjuvants in clinical trials represents an exquisite translation of such preclinical work. However, one could argue that the highly effective preclinical approach of generating and priming subcutaneous tumors has been only partially translated to clinical trials. This is because the subcutaneous tumors in animals during preclinical studies have merely been viewed as expedient surrogates for the primary tumor, which will be irradiated in humans during clinical trials. This is understandable because patients already have primary tumors and there is little rationale to generate additional subcutaneous tumors on patients to serve as the primary tumor.

**Figure 3:**
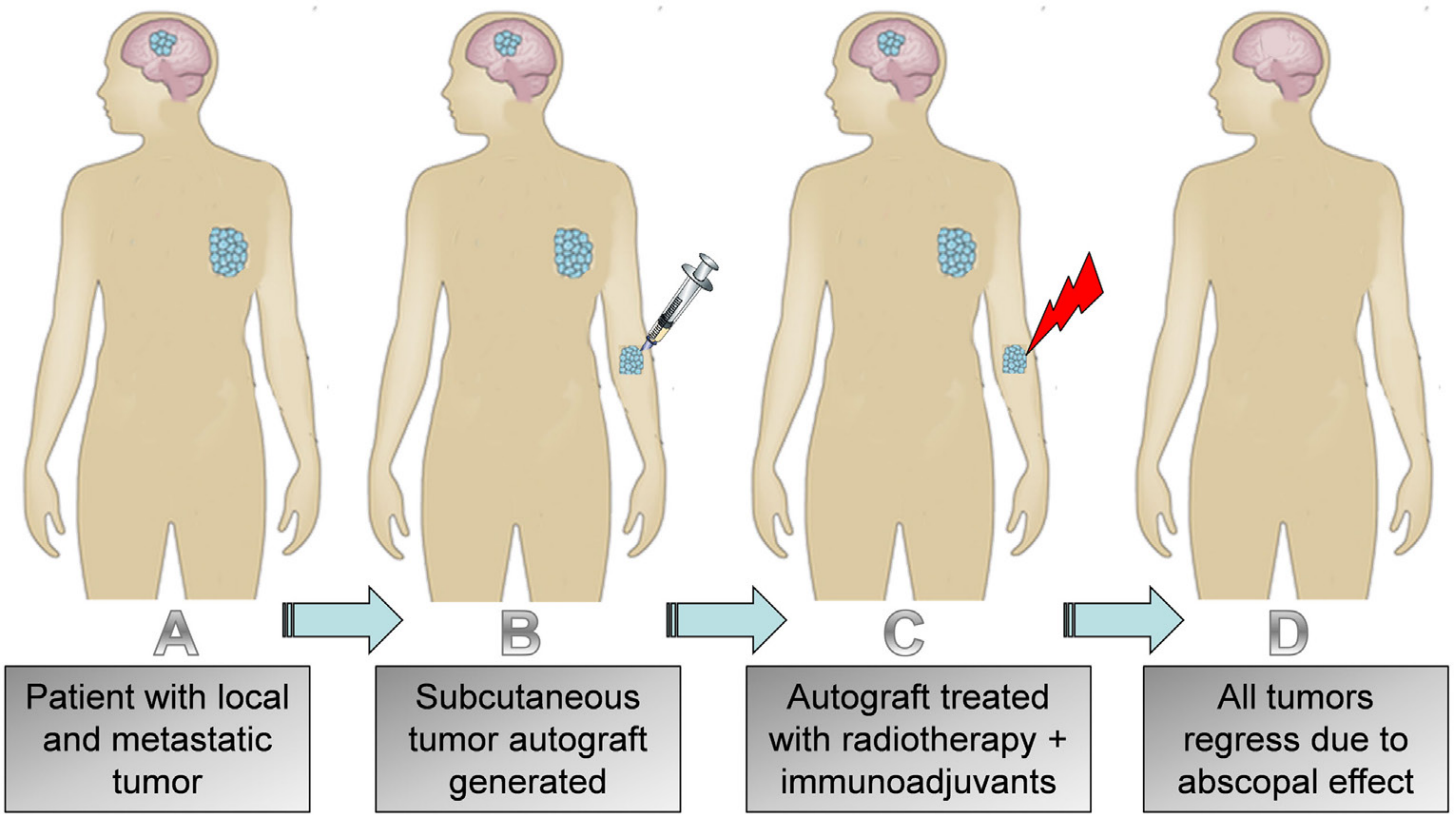
**Illustration of (A) patient with local and metastatic tumor; (B) subcutaneous autograft generated; (C) treatment of subcutaneous tumor with radiotherapy and immunoadjuvant; (D) regression of autograft, primary tumor, and metastasis**.

However, if one actually follows the preclinical data (Table 1), a relatively more accurate translation of these studies would be to also first generate a subcutaneous tumor on patients. This subcutaneous tumor on the patient could then be treated as the primary tumor to prime an effective abscopal effect as in preclinical studies (Figure 3). The patient’s original tumors would then instead serve as metastatic lesions. If effective, as suggested by the preclinical trial data, the immune-mediated abscopal effect would lead to regression of the subcutaneous tumor on the patient, along with any other tumors the patient has. So, following the preclinical data (Table 1), there is rationale for considering the use of subcutaneous tumor autografts in clinical trials employing immunoadjuvants with radiotherapy. However, more rigorous testing in preclinical studies may be needed to optimize such clinical trial planning.

### Better Control of the Priming Process

Another reason for considering subcutaneous autografts in human trials is the potential for greater control of the priming process. The use of subcutaneous tumors provides an opportunity to begin irradiation when the tumor size and associated microenvironment is optimal for priming the abscopal effect. Studies have suggested that the state of the tumor microenvironment and time of treatment is a factor in determining whether an effective abscopal effect is generated (4, 25). The use of subcutaneous models in human trials could afford more control, allowing to begin priming when optimal. Greater control also hearkens to better predictability toward treatment planning.

And with respect to treatment planning, use of subcutaneous autografts will also allow for choosing a convenient location to generate the tumor, farther away from more sensitive neighboring organs at risk (OAR). For example, instead of having to prime the abscopal effect using a lung tumor, which is near neighboring OAR like the heart, one could use a conveniently located subcutaneous tumor on the limbs (Figure 3). This may also be important for patients who need salvage radiotherapy but who have reached their neighboring OAR toxicity limitations, perhaps due to prior radiotherapy treatment. It would also allow for administering radiotherapy to a target where lymphopenia can be avoided (27). Furthermore, a convenient target would allow for hypofractionation, thus effectively reducing treatment times for patients (9, 28). Reducing the treatment times could also help with reducing costs. This is supported by recent studies (29) showing that the use of hypofractionation results in a significant reduction in the financial costs associated with treating breast cancer patients. Such a development will have major impact on the lives of millions of individuals living in low- and middle-income countries and other resource poor settings, who sometimes have to wait months to have access to radiotherapy treatment (28).

Using subcutaneous tumors may also provide more degrees of freedom for engineering the tumor microenvironment to make it more optimal for priming a robust or more efficacious abscopal effect. One could, for example, more conveniently use higher linear energy transfer (LET) radiation to make the tumors more immunogenic. Cancer immunogenicity is described as the ability of a tumor to induce an immune response. It is widely believed that tumor immunogenicity increases with the rate of mutations. The more mutations a tumor has during radiotherapy, the higher the chance that neoantigens can trigger an immune response. Bladder cancer, lung cancer, and melanoma are among the cancers with the highest rate of mutations and seem to have seen the highest abscopal response rates when combining radiotherapy and immunoadjuvants. Radiobiology indicates that certain types of radiotherapy beam qualities like high-LET radiation can generate more mutations in cancer cells. The use of subcutaneous tumors may provide opportunities to use more of such high-LET beams, which are less penetrating, to make tumors more immunogenic, hence potentially resulting in increased abscopal response rates.

In some preclinical studies showing abscopal effects, immunoadjuvants were administered *via* daily repeated injection in the subcutaneous tumors over many days after local radiation therapy (30). The use of subcutaneous tumors in human trials may make it easier to administer the immunoadjuvants, directly into the tumor even repeatedly with minimal systemic toxicities. This may particularly avail the use of immunoadjuvants like GM-CSF or anti-CD40, which enhance the recruitment and activation of APCs within the tumor. Furthermore, more potent immunoadjuvant combinations could be directly administered to the subcutaneous tumor to prime a robust immune response with minimal systemic toxicity. Combinations of immunoadjuvants have been shown to be more effective in immunotherapy but have been limited by systemic or overlapping toxicities reported in clinical trials (9).

The priming of antigen-specific effector T cells is driven by proper antigen presentation and co-stimulation by APCs (31). Since APCs are localized, among other locations, in peripheral tissues such as the skin, the use of subcutaneous tumors may allow for targeting neoantigens to APCs where they are highly populated. Optimal subcutaneous APC targeting in combination with adequate adjuvant delivery may facilitate APC maturation and enhance antigen cross-presentation or T cell priming.

The use of subcutaneous tumors also provides an excellent opportunity to ensure adequate sustained immunoadjuvant delivery with minimal systemic toxicity by employing skin implantable biomaterials for sustained delivery of the immunoadjuvants. Examples of such biomaterials are smart radiotherapy biomaterials (32) (Figure 1) including microneedle versions loaded with immunoadjuvants for sustained *in situ* delivery as highlighted in recent studies (33). Studies have shown that sustained delivery of a vaccine using microneedles elicits increased proliferation of antigen-specific CD8^+^ T cells compared to injections (34). The delivery of immunoadjuvant using skin implantable biomaterials with controlled release over many days is, therefore, expected to also prime a more robust and predictable immune response, consistent with previous work from vaccine studies. So altogether, compared to other approaches, slow *in situ* release of immunoadjuvants would help minimize systemic toxicities and is expected to be more effective in priming an abscopal response (35).

## Demerits for Employing Subcutaneous Autografts

The approach to prime an abscopal effect using subcutaneous tumor autografts is tantamount to first giving a cancer patient more cancer in order to treat the patient. Such an approach is a challenge in at least two ways. First, there is a psychological challenge that must be overcome in explaining to patients why they will get more cancer first. However, if such an approach is shown to be more efficacious in human trials, the negative perception could be assuaged in the longer term. The location of the subcutaneous tumors could also be chosen where it can be effectively treated without significant additional burden to the patients. The strategic choice of location is one way used in surgery in research using tumor homografts (36).

Another thing to consider is that subcutaneous tumors do not fully recapitulate the tumor microenvironment. More studies designed to compare responses for orthotopic and subcutaneous tumors in priming effective abscopal responses may be needed to better address this concern.

In considering the use of subcutaneous tumors in human trials, patient selection could also be the key. It may be advisable to start with patients with advanced or terminal metastatic disease. It is actually expected that these are the patients who could initially get the most benefit from this approach. So, clinical trials employing autografts would have to consider patient selection carefully. Apart from considering patients with advanced disease, the choice of tumor site may be a factor. Subcutaneous metastasis is rare but has been reported in some case studies (37–39). It may be worthy first testing in such patients who already have subcutaneous metastasis.

Another challenge is that patients may reject autografts. In a study by a number of authors, they concluded that patients with already advanced disease showed less rejection of subcutaneous tumors compared to normal people (40). For example, in a study by Southam and Moore, normal recipients responded to implanted cancer cells with a marked local inflammatory response and rapid complete regression of the implants in a maximum period of 3–4 weeks. However, in striking contrast, recipients who had advanced cancer showed little or no acute inflammatory response. Many patients failed to reject implanted cancer cells over periods of observation (40).

There is also a logistical question about how to get cells for the autograft. For many tumors, this could be obtained at time of biopsy or by fine needle aspiration of tumor tissue. In considering this logistical question, the possibility of using subcutaneous tumor homografts also arises. More investigations would be needed to see if one could employ homografts. Ultimately, the risks and benefits of subcutaneous tumors will need to be adequately balanced (Table 3).

**Table 3:** **Merits and demerits of employing subcutaneous tumor autografts to prime a more efficacious abscopal effect**.

Merits	Demerits
Most preclinical studies demonstrating effective abscopal responses have employed subcutaneous models The skin layers are known to be highly populated with professional antigen-presenting cells, which play an important role in effectively inducing abscopal responses. There may be better control of the priming process when using subcutaneous tumors, since priming could be done at optimal tumor sizes or time points, etc. Radiotherapy treatment planning for priming the subcutaneous tumors should be easier if location is chosen distant from sensitive organs at risk. There is an opportunity to use smart biomaterial skin implants for sustained delivery of immunoadjuvants toward more effective treatment outcomes as seen in vaccine studies Benefits of this approach may outweigh the risks for certain groups of patients	Subcutaneous tumors are expedient but provide limited recapitulation of the tumor microenvironment There is a need to first give patients an additional lesion before treating them Patients may reject autografts or homografts

## Further Discussion

Other than using subcutaneous tumor autograft, there are some differences between the preclinical and clinical studies that may affect the treatment outcomes. For example, mice radiotherapy is typically done in a very different way than that of humans for the beam quality, field size, fractionation, radiation dose, etc. Some of these factors may not be adopted into the clinical studies. Others, when being adopted, may benefit from the flexibility provided by the subcutaneous tumor autograft.

There are ethics concerns involved in the new proposed treatment scheme. Careful design of the clinical trials is necessary. The baseline of medicine and medical research is to act in the patient’s best interest (41). Medical research that involves human subjects should ensure safety, effectiveness, monitorable procedure, and predictable results; minimize the patient’s risks and burden; and provide the best possible care compared to all other alternatives (42). Based on these guidelines, clinical research for studying the subcutaneous tumor autograft may follow some recommendations as described in this section.

First of all, patients with terminal diseases may benefit from this treatment the most. To start with, the treatment may be tested in patients with subcutaneous metastasis (37–39, 43). In this case, there is no need for generating an autograft. Next, one would recommend continuing the study with stage IV none small cell lung cancer (NSCLC). Overall, lung cancer is the leading cause of cancer death for both men and women in the United States (44). For stage IV NSCLC patients, the 5-year survival rate is less than 10%, and the current treatment recommendation is to use chemotherapy. With the proposed treatment, if the abscopal effect is induced, the treatment outcome may exceed the current available options. Besides, previous studies (8) (Table 1) and current clinical trials (Table 2) with lung cancer patients provide valuable knowledge for research design.

Second, the patient’s risks and burden should be evaluated. With the current survival rate for stage IV NSCLC patients—less than 10% 5-year survival, which means the probability of death due to the cancer is close to 100%, it is almost impossible to increase patient’s risks. In fact, preclinical results are promising, and good chances of increasing the survival rate are expected. Furthermore, the proposed local treatment should minimize systemic toxicity and reduce the patient’s burden. However, statistics for the entire patient population should never be confused with each individual case. For the actual clinical study, patients must be evaluated individually by their physicians and the clinical researchers.

Throughout the treatment, patients should be monitored closely for their response. The treatment site or the autograft can be easily measured for its response to radiotherapy. Imaging modalities, like CT and PET, can be used to assess the abscopal response. As reported by Golden et al., significantly lower neutrophil to lymphocyte ratio is presented in patients who have abscopal responses (8). Other methods, like T cell trafficking (27), may also be useful for treatment monitoring.

## Conclusion

Given the significant body of preclinical work showing the effectiveness of subcutaneous models in generating the abscopal effect, more preclinical studies designed to better assess the risks of generating subcutaneous autografts in clinical trials should first be considered to provide more data. Such studies could involve comparison with orthotopic tumor models. If the use of subcutaneous autografts is further justified by such data and validated, the impact of such an approach would be significant. It would further extend the use of radiotherapy to the treatment of both local and metastatic disease. Metastasis accounts for over 90% of all cancer-associated suffering and death, hence, such an approach would be of great benefit to many cancer patients.

## Author Contributions

WN proposed the concept, made substantial contribution to the work, and wrote the manuscript. ZO assisted in the research and edited the manuscript. Both authors approved the manuscript.

## Conflict of Interest Statement

The authors declare that the research was conducted in the absence of any commercial or financial relationships that could be construed as a potential conflict of interest.
